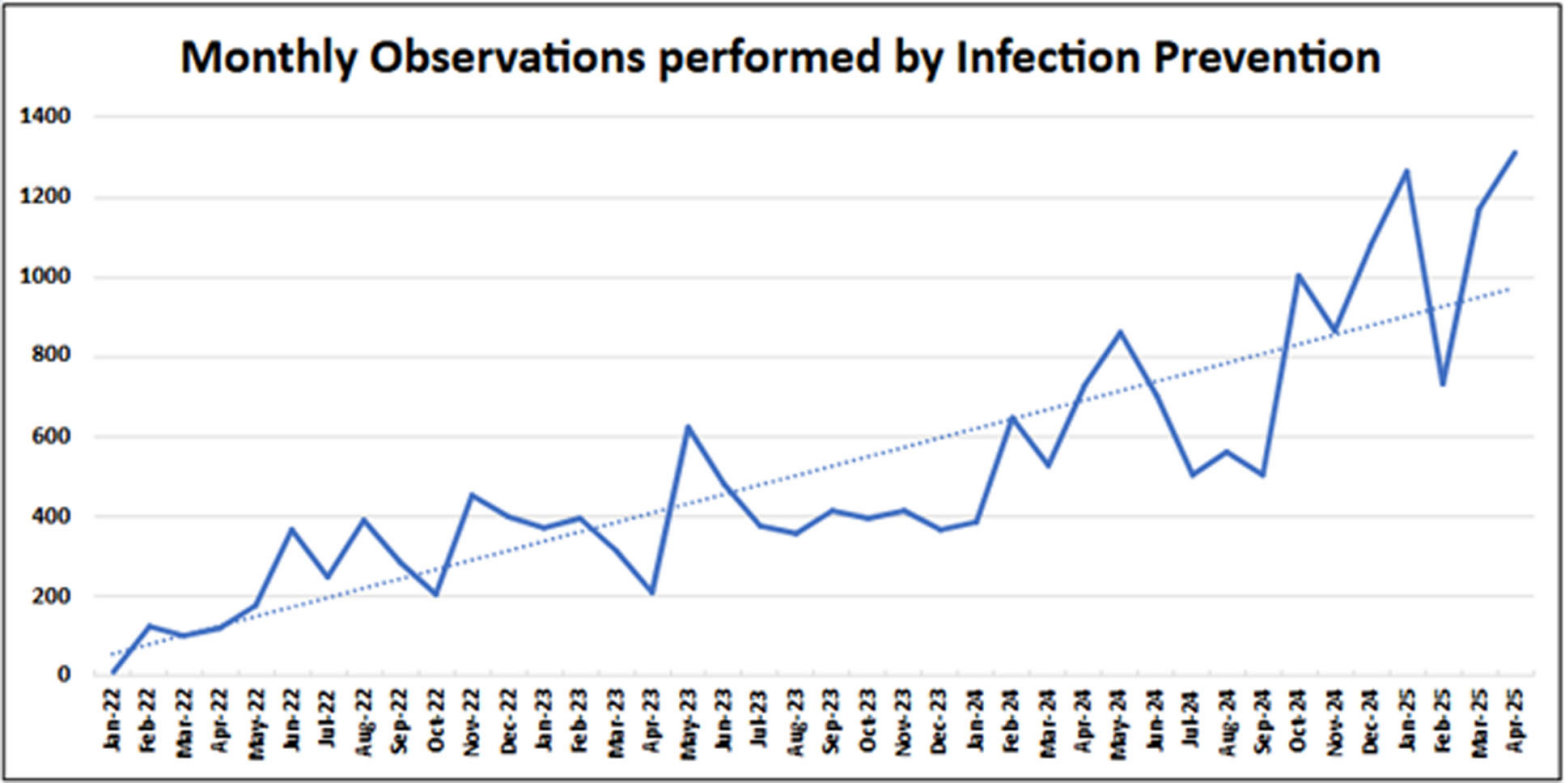# 258 Candida auris Surveillance: Developing a Predictive Model to Optimize Early Identification

**DOI:** 10.1017/ash.2026.10625

**Published:** 2026-06-23

**Authors:** Ryan Penn, Marci Drees, Serena Wingel

**Affiliations:** 1 ChristianaCare

## Abstract

**Background:** Hand hygiene is the most effective way to prevent hospital acquired infections, and thus evaluating hand hygiene compliance is critical to ensure patient safety. The Leapfrog Group is a non-profit organization that, in part, evaluates organizations' commitment to hand hygiene as a component of their overall hospital safety grades. For those performing manual observations, Leapfrog requires most inpatient units to complete at least 200 observations per month to achieve an "A" grade. At our facility, hand hygiene observations are performed manually by trained unit-based caregivers as well as by Infection Preventionists (IPs) who serve as “validators.” Between January 2022 and June 2023, on average only 37.4% of inpatient units met their Leapfrog goals; IPs (n=10) performed an average of 294 validation observations per month. Our goal was to improve organizational commitment to hand hygiene compliance, increase IP validator rounds, and achieve <95% of units meeting their monthly Leapfrog goals systemwide throughout FY24 and beyond. **Methods:** ChristianaCare is a 3-hospital, <1400-bed community-based academic healthcare system based in northern Delaware. In March 2023, leadership committed to meeting and maintaining established Leapfrog goals for hand hygiene observations for inpatient units systemwide. Nursing leadership, in conjunction with IP and the Hand Hygiene Steer Committee, became actively involved with monitoring and meeting these goals. The Hand Hygiene Steer Committee initiated active conversations with individual units, as well as established real-time reporting of progress towards 95% goal to units. IP also initiated a robust recognition program, recognizing units as well as individual caregivers for their successes in hand hygiene observations and meeting goals. **Results:** Between Oct 2022-June 2023, the percentage of units meeting their Leapfrog observation goal rose from 37.4% to 96.2% (Figure 1). From July 2023 onwards, the average Leapfrog goal compliance for inpatient units was 96.2% (a 58.8% increase; Figure 2). In addition, the average monthly number of observations performed by the Infection Prevention Department was 690 (a 235% increase). A total of 8 inpatient units and 7 individual caregivers were recognized systemwide for their hand hygiene efforts, successes, and improvements. **Conclusion:** Maintaining hand hygiene compliance and a systemwide hand hygiene observation program requires commitment and significant effort throughout the organization. Continued monitoring, ongoing discussions, and targeted interventions have demonstrated ChristianaCare’s ability and commitment to hand hygiene and meeting The Leapfrog Group’s goals for patient safety.

**Figure:**
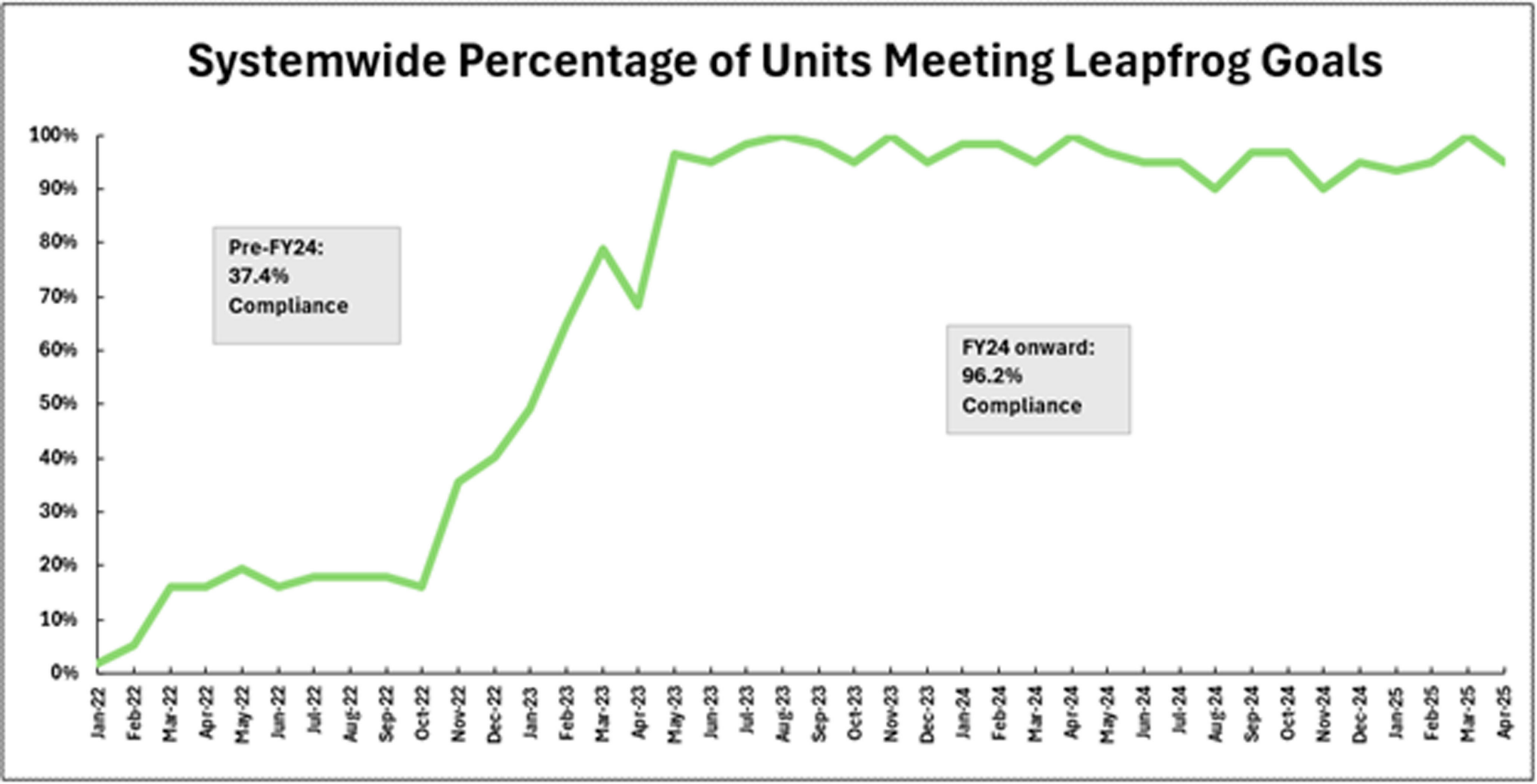

**Figure:**